# COVID-19 surveillance in the Flemish school system: development of systematic data collection within the public health school system and descriptive analysis of cases reported between October 2020 and June 2021

**DOI:** 10.1186/s12889-022-14250-1

**Published:** 2022-10-15

**Authors:** Joanna Merckx, Jonas Crèvecoeur, Kristiaan Proesmans, Naïma Hammami, Hilde Denys, Niel Hens

**Affiliations:** 1 Department of Epidemiology, Biostatistics, and Occupational Health, McGill University, 2001 McGill Street, Suite 1200, Montreal, QC H3A 1G1 Canada; 2 I-BioStat, Data Science Institute, Hasselt University, Hasselt, Belgium; 3 Department of Epidemiology and Public Health, Sciensano, Brussels, Belgium; 4 Agency for Care and Health, Infection Prevention and Control, Flemish Community, Brussels, Belgium; 5 Department Onderwijs, Brussels, Belgium; 6 Centre for Health Economic Research and Modelling Infectious Diseases, Vaccine and Infectious Disease Institute, University of Antwerp, Antwerp, Belgium

**Keywords:** Surveillance, Schools, SARS-COV-2, Children, Cases, Epidemiology, Testing

## Abstract

**Background:**

The age-specific distribution of SARS-CoV-2 cases in schools is not well described. Reported statistics reflect the intensity of community transmission while being shaped by biases from age-dependent testing regimes, as well as effective age-specific interventions. A case surveillance system was introduced within the Flemish school and health-prevention network during the 2020–2021 school year. We present epidemiological data of in-school reported cases in pre-, primary and secondary schools identified by the case surveillance system, in conjunction with test data and community cases from October 2020 to June 2021.

**Methods:**

We describe the development of the surveillance system and provide the number of reported cases and standardized rates per grade over time. We calculated absolute and relative differences in case incidence according to school grade (primary: grades 1–6, and secondary: grades 7–12) using grades 7–8 as a comparator, relating them to non-pharmaceutical infection prevention interventions. Cumulative population incidences (IP) stratified by age, province and socioeconomic status (SES) of the school population are presented with their 95% confidence intervals (CI).

**Results:**

A total of 59,996 COVID-19 cases were reported in the school surveillance system, with the highest population adjusted IP in grade 11–12 of 7.39% (95%CI 7.24–7.53) and ranging from 2.23% to 6.25% from pre-school through grade 10. Age-specific reductions in mask introduction and in-person teaching were temporally associated with decreased case incidence, while lower pupil SES was associated with an increase in cumulative cases (excess 2,739/100,000 pupils compared to highest SES tertile). Community testing volumes varied more for children compared to adults, with overall higher child test-positivity. Holidays influence capturing of cases by the system, however efficiency increased to above 75% after further automation and integration in existing structures.

**Conclusion:**

We demonstrate that effective integration of case surveillance within an electronic school health system is feasible, provides valuable data regarding the evolution of an epidemic among schoolchildren, and is an integral component of public health surveillance and pandemic preparedness. The relationship towards community transmission needs careful evaluation because of age-different testing regimens. In the Flemish region, case incidence within schools exhibited an age gradient that was mitigated through grade-specific interventions, though differences by SES remain.

**Supplementary Information:**

The online version contains supplementary material available at 10.1186/s12889-022-14250-1.

## Introduction

In-person learning in primary and secondary schools is at the center of the educational system internationally, and is broadly considered the optimal environment for intellectual, personal and social development of children and teenagers [1]. However, prolonged contact between large numbers of school children and teachers facilitates the spread of infectious diseases by airborne, droplet and contact transmission [2, 3]. Since the start of the COVID-19 pandemic, SARS-CoV-2 infection and transmission in the school environment have been under large scrutiny. Efforts to better characterize COVID-19 transmission dynamics within pediatric populations have suffered from a lack of data and potential biases due to selective collection of data [4], as with testing regimens that focus on symptomatic individuals only [5]; this is particularly true for research which aims to explore transmission dynamics among school-age children receiving in-person education. In spite of these challenges, systematic surveillance of cases in the school setting remains a useful method of describing and quantifying changes in transmission dynamics, by age and over time [6].

The COVID-19 pandemic is a public health emergency affecting individuals of different ages differently. Pediatric cases are generally mild and not captured through hospitalization or health care visit data [7, 8]. Analyses of data collected from admitted children produces biased estimates of infection rates in the pediatric population. Several outbreaks have been documented in school settings, with infection and transmission occurring among pupils and staff alike [9, 10]. The role of in-school transmission and the interaction with community and household transmission requires additional scrutiny using quantitative epidemiological tools and data collection within the school environment, while linking with community data [11]. A solid surveillance system that can be set up and used during epidemics can be a resourceful tool to measure the impact of interventions and to timely inform decision making.

In Belgium, schools were gradually and partially re-opened in the spring of 2020 as part of the exit strategy following the first wave and corresponding lockdown. All primary and secondary schools, both from the Flemish and French language school system, resumed full-time in-person education on September 1^st^, 2020. Flemish schools are under the responsibility of the Ministry of Education of the Flemish region and all public and private schools that are approved, financed, or otherwise subsidised by the Flemish government are connected to the school public health system directly. The network is primarily organized via Student Guidance Centers known as Centra voor Leerlingenbegeleiding (CLBs). Within this structure and with the help of the Belgian national public health agency, Sciensano, and the regional public health agency Vlaams Agentschap Zorg en Gezondheid (VAZG), a COVID-19 school surveillance system was set up in September 2020 with occasional adjustments during the school year. Parallel, in the French region, a similar system was developed with separate data collection and supervision, with final aggregation of all collected data by Sciensano.

The aims of this manuscript are to (i) describe the development of a surveillance and testing-and-tracing system for COVID-19 cases and potential school transmission in Flemish pre-, primary and secondary-schools and to (ii) describe the frequency and epidemic curve of SARS-CoV-2 confirmed school cases by age group from October 2020 to June 2021 in conjunction with non-pharmaceutical interventions (NPI) implemented to control transmission in schools, the community background incidence and national age-specific test data, using data obtained from the school surveillance database.

## Methods

### Study design and study population

This study reports on the design and implementation of a surveillance system of SARS-CoV-2 confirmed and reported cases in all children attending public and governmental-supported pre-, primary and secondary schools of the Flemish Community in Belgium, from October 5^th^, 2020, until June 27th, 2021. Special education schools and their pupils are not included in the study.

The Flemish school system is organized into three publicly funded educational networks and has 2,454 pre- and primary schools and 948 secondary schools [12], with a total of 1,106,194 registered pupils as of February 2020 [13] (Figure S1). The school health network consists of 58 independent Student Guidance Centers (CLB’s), including medical doctors and nurses responsible for preventive health care, and is under the responsibility of the Flemish government. Data on confirmed and reported SARS-CoV-2 cases among school personnel was not consistently available during the surveillance period and is therefore not included in this study.

### Background non-pharmaceutical infection prevention and control interventions (NPIs) with implications for schools

**Fig. 1:**
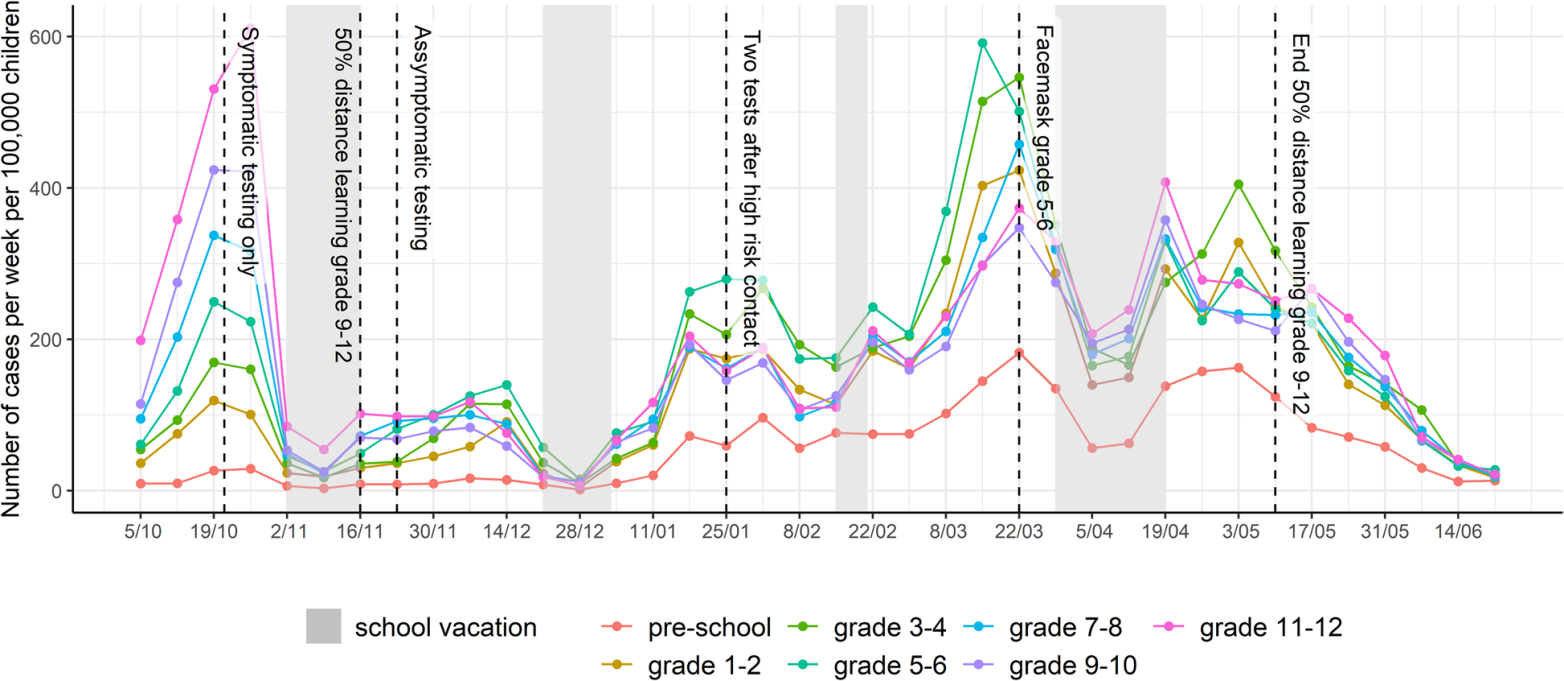
Evolution of the number of reported COVID-19 cases per week per 100,000 children and school-related NPI. Legend: NPI: non-pharmaceutical interventions. Pre-school: age 2.5-5yo, grade 1-2: 6&7 yo, grade 3-4: 8&9 yo; grade 5-6: 10&11 yo; grade 7-8: 12&13 yo; grade 9-10: 14&15yo; grade 11-12: 16&17yo; yo: years old, intended age of pupils on January 1th while in the corresponding grade

When schools were opened on September 1st, 2020, with in-person education for all pupils of all grades, masks were introduced from grade 7 and up to be worn in class and outside class, when maintaining a distance of at least 1.5 m was impractical; as for all adult personnel in all school institutions, except for those having contact with pre-school children. Physical distancing between adults was encouraged, however not enforced between pupils. Hand hygiene was promoted. Class bubbles were not kept separate in most elementary schools [14] and no changes in class size were introduced. The pre-scheduled one-week fall break started on October 31^st^, 2020 for all pupils, and was ultimately extended until November 15th, 2020. Preschools and primary schools were fully re-opened on November 16th, while part-time in-person education and part-time home education for all pupils from grade 9 and up was initiated, resulting in an overall 50% attendance (either with or without reduction of class sizes which was decided on school level) and non-mixing of class bubbles. The winter break occurred during the originally scheduled period (week of December 21st, 2020, until January 4^th^ 2021). The week of March 22^nd^, 2021, mask wearing was introduced in class for pupils in the 5^th^ and 6^th^ grades. The Easter holidays were advanced by one week, defined as suspension of all education institutions between March 29^th^ and April 2^nd^, except continuation of in-person examinations, resulting in a total duration of the April vacation of three weeks for elementary schools. Key school-related NPIs are represented in Fig. 1. Additional details can be found in the Supplement.

Diagnostic testing for confirmation of acute SARS-CoV-2 infection during the 2020–2021 school year was mainly performed using PCR tests, with only limited use of antigen tests in the period under investigation, and is captured in the national surveillance system of Sciensano [15, 16]. National testing strategies changed between October 21st and November 23rd, 2020, due to test capacity issues in conjunction with a large surge in community cases. During this period only individuals suspected of having COVID-19 disease based on reported symptoms were tested. After November 23^rd^, testing of all potentially infected cases and of those with high-risk contact (HRC) was re-introduced. As of January 2021, large testing campaigns were initiated in schools, mainly driven by the increase of the alpha variant in the community. Testing frequency was also intensified for HRC after January 25^th^, 2021, with two recommended test moments after identification of the contact [17]. Contact tracing was performed in schools using the described surveillance system, with differential measurements of quarantine for high versus low-risk contacts and age-dependent (e.g., less than versus more than 6 years old) and NPI-dependent (e.g. mask versus no mask on exposure) assignment of risks and consequential testing.

### Data sources

For the analysis, anonymized data from the surveillance network were transferred on a biweekly basis to the study team, starting with the first data collection using the automated platform LARS (Leerling Activiteiten en Registratie Systeem) (Week October 5th, 2020). In addition, we used age-stratified community-reported test data from the national COVID-19 laboratory network surveillance as centralized by Sciensano. These data are available in real-time for the regional public health services, including PCR and antigen tests performed in Flanders, by age, date of sampling and test result [16].

Population numbers by age and municipality were extracted from Statbel [18] for the denominators. Total count of pupils by school level and school are based on 2019–2020 academic year data [13]. An overview of NPIs in place at the school and community level, by time of implementation, was provided by the Department of Education. Socioeconomic pupil indicators by school, publicly available for financial and resource allocation in the Flemish school system [19], were used to construct a summary socioeconomic status (SES) variable, including maternal education, language spoken at home, pupil subsidy for education and neighborhood’s delay in schooling.

This study provides aggregate anonymized data. No ethical approval was needed for the analysis and the data were provided by the Ministry of Education and Sciensano to the study team in accordance with a data agreement for the age-stratified community test data.

### Data analysis

We provide a descriptive analysis. Cases captured in the surveillance system are presented in absolute numbers and as weekly (Monday to Sunday) incidence proportions per 100,000 population by school grade and by time. Absolute and relative differences in age-stratified case incidences between ages are presented longitudinally in figures. Crude absolute cumulative incidence numbers by grade, province and SES level are presented in tables with their 95% confidence intervals (CI). Expected and observed number of cases are compared by SES using binomial tests. A supplementary analysis investigates (i) case increments over time versus tests in the community in children and in adults and (ii) adult versus child test positivity rates using plots. Statistical analysis was performed in R version 4.0.2 (R Foundation for Statistical Computing, Vienna, Austria, 2020) and STATA 13 (Statcorp. College Station, TX).

### Role of funding

The surveillance system is under the responsibilities of the Ministries of Education and Health and receives no additional or specific funding. The investigators involved in the study did not receive separate funding for the analysis. All investigators had access to the anonymized and secured surveillance data.

## Results

### Development of the surveillance network

The surveillance system was set up at the start of the new school year on September 1st, 2020, within the Flemish government-aided education school network, nested within the school health network (CLB) and guided by VAZG and Sciensano. All children who tested positive with a Polymerase Chain Reaction (PCR) test on an upper respiratory tract sample and who physically attended the school were entered in the surveillance system as individual laboratory-confirmed cases. It is the responsibility of the school to report confirmed cases to their corresponding CLB, which then performs an investigation of the case through contact with the school, pupil and his/her parent or guardian. Investigation entails contact and contact risk assessment in the school environment and, if necessary, prescription of testing. General infection prevention and control guidelines were prepared by the Ministry of Education and guidelines for testing, contact tracing and quarantine were drafted based on the advice of Sciensano and in coordination with VAZG. When a cluster is detected in a school, i.e., two or more potentially related cases within a period of 14 days, the CLB then contacts the local COVID-19 team or regional public health unit VAZG, for registration of the outbreak and if necessary, further in-depth analysis, screening and contact-tracing embedded within the national contact tracing network. CLB clinicians and personnel are bound to patient confidentiality and secure collection of all information. Data was initially collected using spreadsheets completed by the CLBs and collected by the Ministry of Education and Sciensano. As of October 5^th^, 2020, surveillance data are entered, tracked and can be shared through the secured school health automated platform LARS which secures availability of pupil demographic data. As of November 22^nd^, 2020, this was the only data registration pathway. Data is extracted and compiled to anonymized datafiles by each CLB and transferred biweekly to the Ministry of Education, for policy guidance at the ministry level, and also to the regional public health services and Sciensano. Sciensano publicly reports the data together with school data from the French language school network (Brussels and Wallonia) in the weekly epidemiologic bulletin section ‘Situation of COVID-19 in Children’ [20] and in the weekly risk assessment report of Sciensano [21].

Beginning on January 18^th^, 2021, a daily automated data flow was set up between i) the SARS-CoV-2 positive test results available in the community-testing driven data platform of VAZG and ii) the school system, LARS, allowing more time-efficient case detection and management by the CLBs. Contact tracing data from LARS are then transferred back to the data platform at VAZG to inform the regional public health unit about the situation in schools. A flowchart of the data components and streams is provided in Figure S2. Collected variables used in the LARS database and for the data analysis include: name of child, date of registration of a positive case, child’s class and school level, reason for SARS-CoV-2 testing, probable place of infection, secondary cases linked to the index case, number of children and number of personnel in quarantine after registration of the case.

### Cumulative cases

Between October 5^th^, 2020 and June 27^th^, 2021 a total of 59,996 cumulative acute COVID-19 cases among students were reported in the Flemish school surveillance system (Table 1). Pupils from pre-school and enrolled in grade 1–2 had the lowest population adjusted cumulative incidence, 2.23% (95%CI 2.17–2.29) and 5.23% (95%CI 5.11–5.34), respectively. Cumulative incidences in pupils from grade 3–4 and up through grade 9–10 were similar, ranging from 6.22% to 6.94%. Grade 11–12 pupils had the highest cumulative incidence of reported cases of 7.39% (95%CI 7.24–7.53). Provincial differences apply, with incidences ranging from 3.62% (95%CI 3.54–3.71) in Flemish-Brabant to 6.85% (95%CI 6.62–7.08) in the Brussels Capital region. Incremental differences are apparent in cumulative incidence of cases by pupil SES score, with significantly fewer cases in schools in the highest SES tertile compared to the middle and lowest SES tertile. We estimated an absolute excess of 2,739 cases per 100,000 pupils attending schools in the lowest SES tertile compared to the highest tertile (Table 1).

**Table 1:** Cumulative number and cumulative percentage of COVID-19 reported cases in the surveillance system

	**Cumulative number of reported SARS-CoV-2 cases**	**Population**	**Cumulative percentage reported (95% CI)**
**School grades Total**	59,996		
pre-school (2.5–5 yo)	5,825	261,131	2.23 (2.17–2.29)
grade 1–2 (6&7 yo)	7,583	145,074	5.23 (5.11–5.34)
grade 3–4 (8&9 yo)	9,419	142,514	6.61 (6.48–6.74)
grade 5–6 (10&11 yo)	9,527	137,311	6.94 (6.80–7.07)
grade 7–8 (12&13 yo)	9,461	152,076	6.22 (6.10–6.34)
grade 9–10 (14&15 yo)	8,883	142,087	6.25 (6.13–6.38)
grade 11–12 (16&17 yo)	9,298	125,879	7.39 (7.24–7.53)
**Provinces Total**	59,996		
Antwerpen	16,574	312,393	5.31 (5.23–5.38)
Brussels Hoofdstedelijk Gewest	3,292	48,077	6.85 (6.62–7.08)
Limburg	7,985	138,495	5.77 (5.64–5.89)
Oost-Vlaanderen	15,726	250,477	6.28 (6.18–6.37)
Vlaams-Brabant	6,291	173,659	3.62 (3.54–3.71)
West-Vlaanderen	10,128	182,971	5.54 (5.43–5.64)
**Socioeconomic** **Status school population Total**	59,573^a^		
SES high	14,995	365,071	4.11 (4.04–4.17)
SES mid	19,572	376,020	5.21 (5.13–5.28)
SES low	25,006	364,981	6.85 (6.77–6.93)

*yo* Year-old, intended age of pupils on January 1st while in the corresponding grade; SES: low = 33% lowest socio-economic pupil indicator scores, mid = 33%-67%, high = 67%

^a^data linkage was not possible for 423 positive cases which are omitted from the SES analysis

### Surveillance system epidemic curve of reported cases by age

**Fig. 2:**
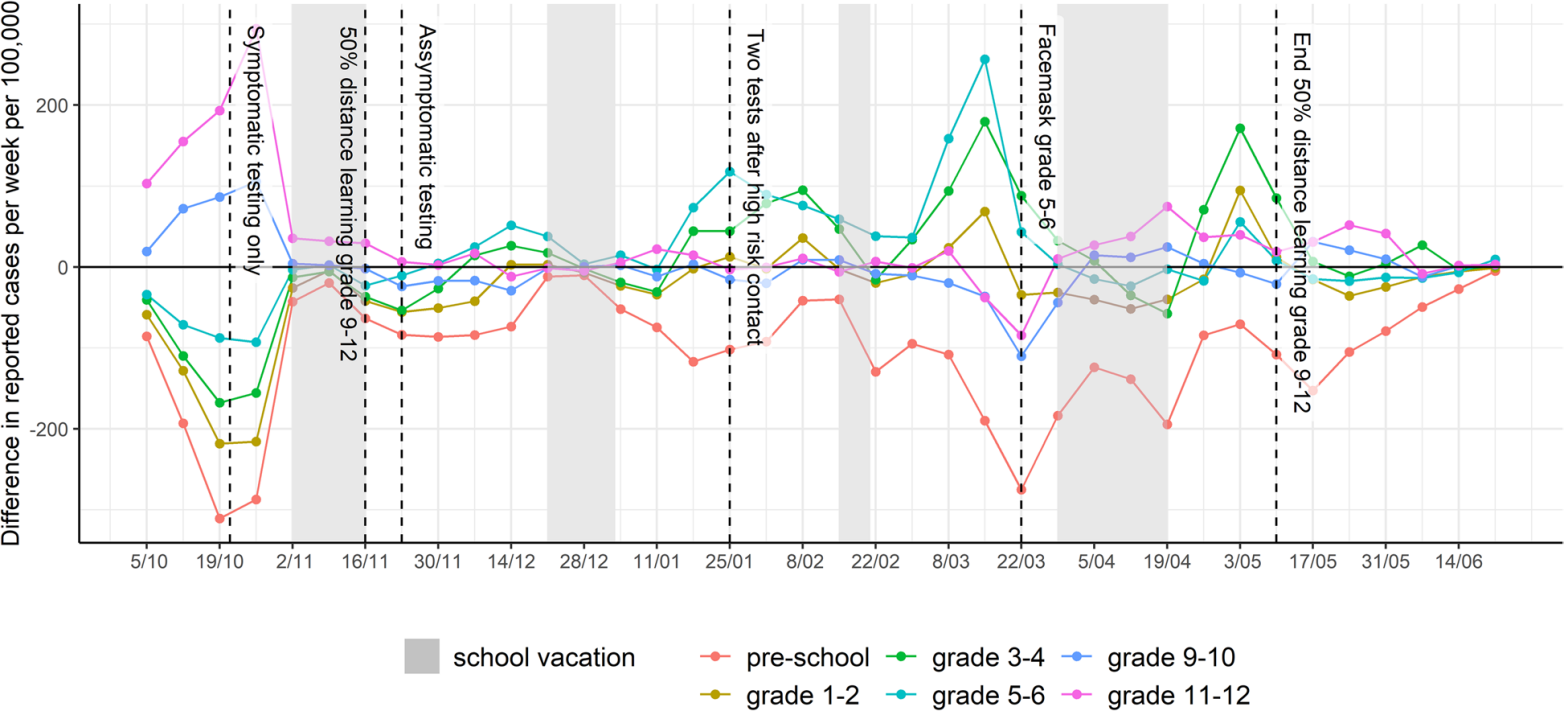
Absolute difference in reported COVID-19 cases per week per 100,000 compared to grade 7–8. Pre-school: age 2.5-5yo, grade 1-2: 6&7 yo, grade 3-4: 8&9 yo; grade 5-6: 10&11 yo; grade 7-8: 12&13 yo; grade 9-10: 14&15yo; grade 11-12: 16&17yo; yo: years old, intended age of pupils on January 1th while in the corresponding grade

**Fig. 3:**
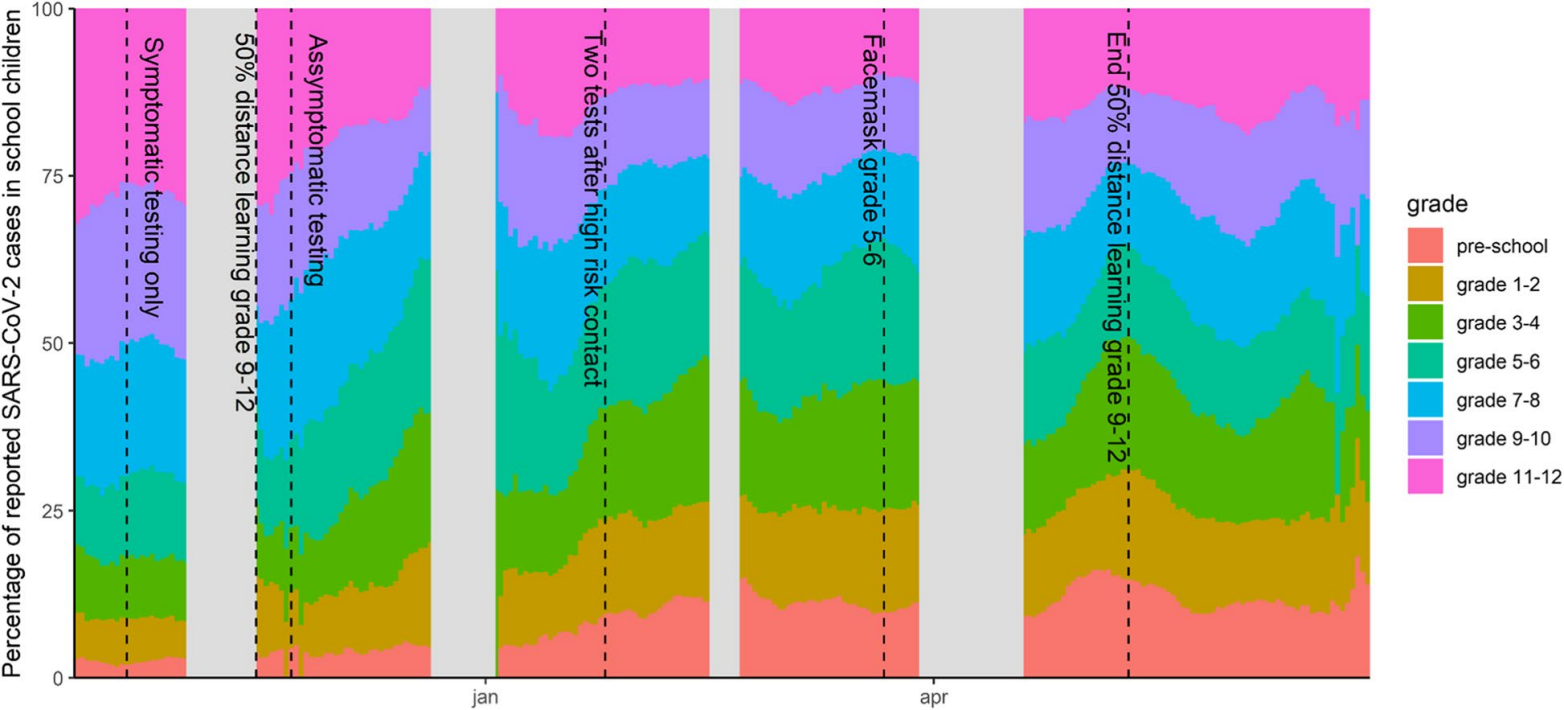
Evolution of the distribution of the percentage of reported COVID-19 cases in school children by grade. Pre-school: age 2.5-5yo, grade 1-2: 6&7 yo, grade 3-4: 8&9 yo; grade 5-6: 10&11 yo; grade 7-8: 12&13 yo; grade 9-10: 14&15yo; grade 11-12: 16&17yo; yo: years old, intended age of pupils on January 1th while in the corresponding grade

The epidemic curve of grade-specific reported SARS-CoV-2 cases per week per 100,000 children is presented in Fig. 1, in which school holiday periods and changes in NPIs are also identified. Figure 2 shows the absolute difference in cases per week per 100,000 by grades, taking grade 7–8 as the reference (relative ratio by grades presented in Figure S3). A positive association between age and case incidence was identified at the beginning of the reporting period; following the initiation of half-time in-person education among students in grades 9 and above, this relationship was no longer apparent. The maximum difference in weekly cases per 100,000 pupils was observed during the week of October 26^th^, when 295 excess cases occurred among grade 11–12 students. The distribution of the proportion of reported cases by grade and time is presented in Fig. 3, which illustrates the evolution of the epidemic given NPIs and testing regimens in effect, and observed decreases in age differences from grade 11–12 to grade 5–6. Grades 5–6 experienced the highest case burden after mid-November 2020, increasing to 256 more cases per week per 100,000 pupils by mid-March 2021 compared to grade 7–8 (Fig. 2). This excess compared to other age groups dissolves temporally after the introduction of masks in grades 5–6.

### Influence of community testing and community cases

**Fig. 4:**
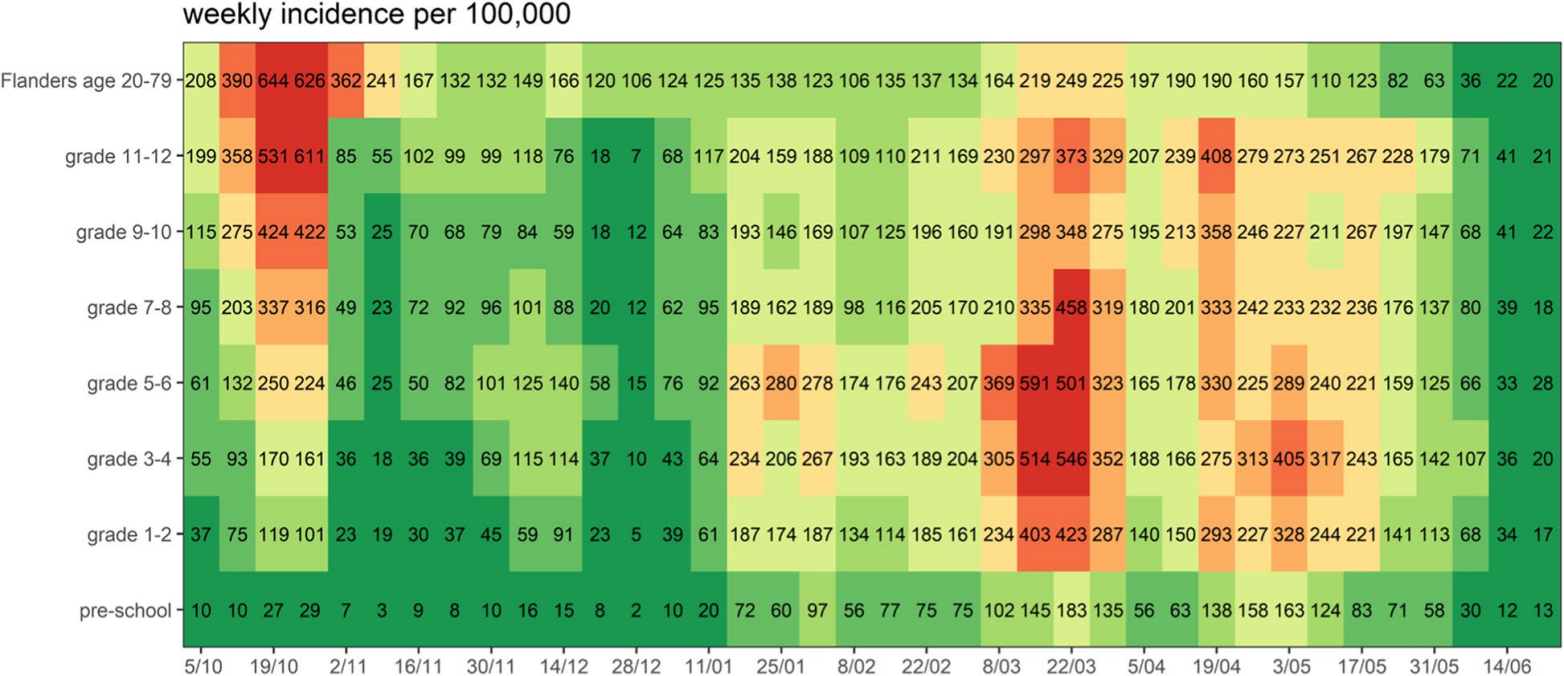
Community test-positive cases in relation with weekly incidence of children captured in the surveillance network. Pre-school: age 2.5-5yo, grade 1-2: 6&7 yo, grade 3-4: 8&9 yo; grade 5-6: 10&11 yo; grade 7-8: 12&13 yo; grade 9-10: 14&15yo; grade 11-12: 16&17yo; yo: years old, intended age of pupils on January 1th while in the corresponding grade

**Fig. 5:**
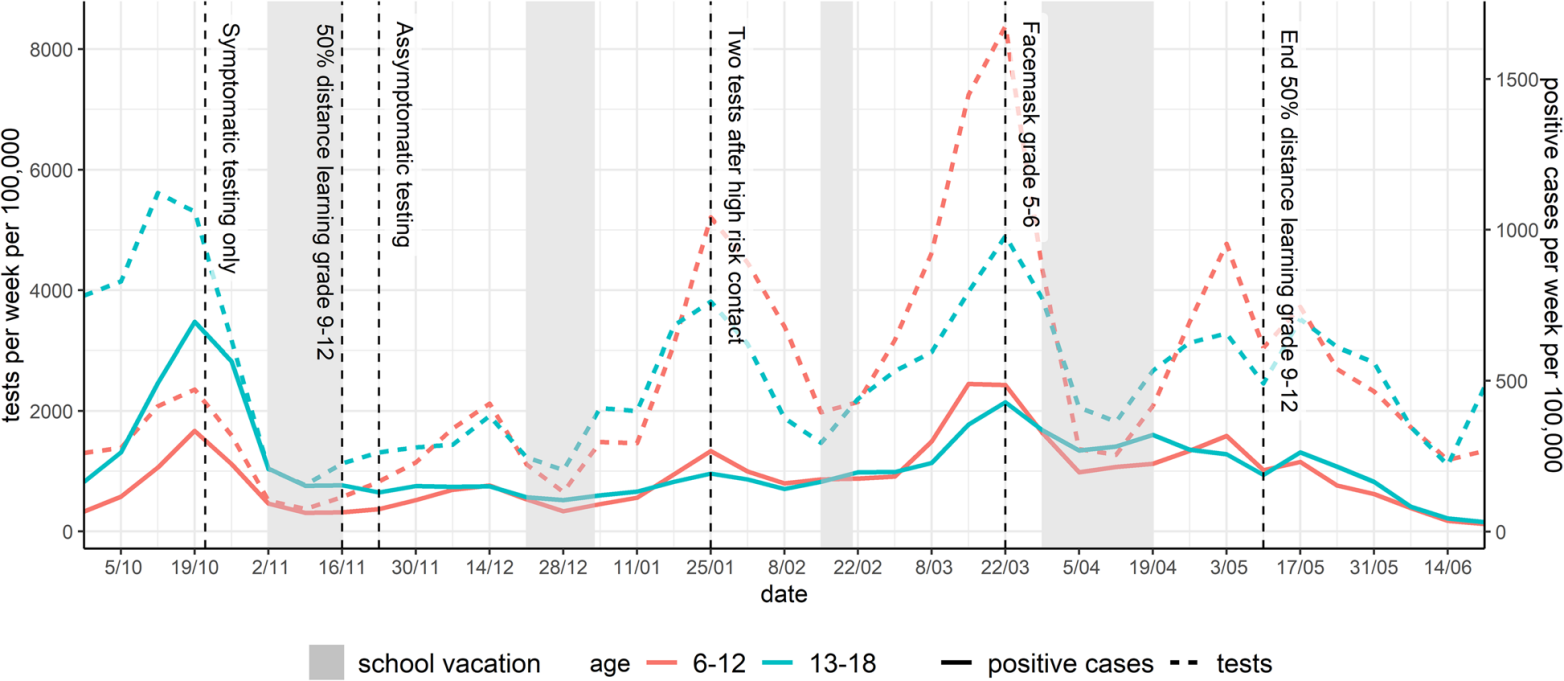
Evolution number of (positive) SARS-CoV-2 PCR and antigen tests in children 6–18 years old in Flanders. Pre-school: age 2.5-5yo, grade 1-2: 6&7 yo, grade 3-4: 8&9 yo; grade 5-6: 10&11 yo; grade 7-8: 12&13 yo; grade 9-10: 14&15yo; grade 11-12: 16&17yo; yo: years old, intended age of pupils on January 1th while in the corresponding grade

**Fig. 6:**
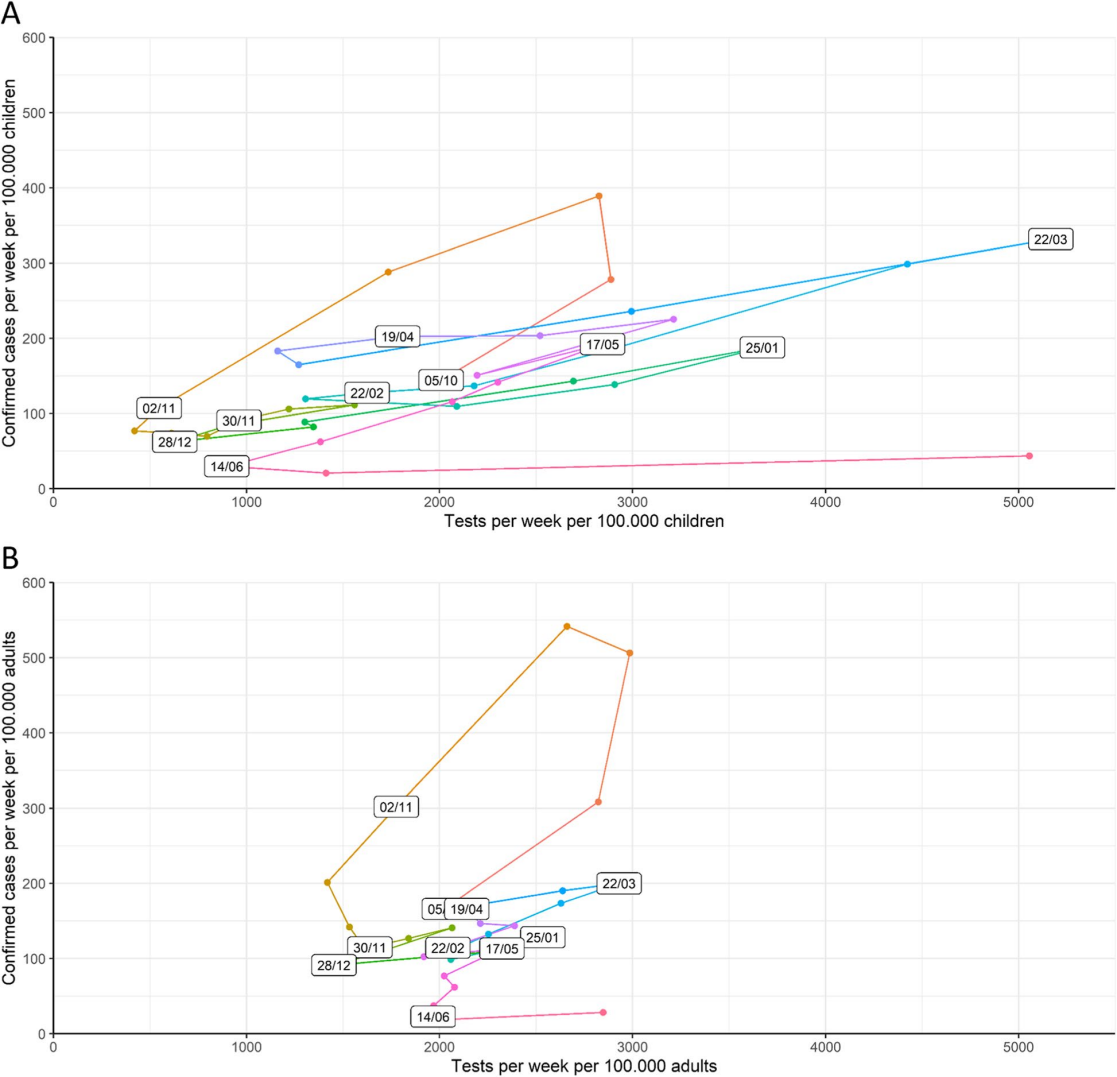
A and B: Number of cases and SARS-COV-2 tests per week in (top) children aged 6–18 and (bottom) people aged 19–80 in the community. Conceptual interpretation of the figures: The figures combine testing volume with test positivity over time and present children and adults separately for their comparison. The X-axes represent tests per week per 100.000 children in the top figure and per 100,000 adults in the bottom figure. Y-axes are confirmed cases per week per 100,000 children in A and adults in B. Calendar time, date/month, is represented with the date in the white squares and is chronologically connected by colored lines. Dates are week end points and colors are similar for the same time periods in adults and children for comparison between graph A and B. Data points in the lower left corner mean a low number of confirmed cases and a low number of tests performed per week per population; right upper corner means high number of confirmed cases and high number of tests performed. Left upper corner means high confirmed case numbers with low testing volume; right lower corner datapoints show that confirmed case count was low, while large number of tests were performed. Over time there is a much larger variation in number of tests performed per population in children compared to adults and tests performed by age group are not correlated in time. In children it is more clear that an increase in tests performed is associated with more confirmed cases in March 2021, while large testing volumes did not catch many confirmed cases in June 2021, at study end

Figure 4 visualizes weekly incidence among community test-positive cases per 100,000 inhabitants aged 20 to 79 versus incident cases in children captured by the surveillance network; time-dependent increases in all age groups were identified. Figure 5 shows the evolution in the number of tests performed in the community and positive tests in children 6- to 11-year-old and 12- to17-year-old over time, with larger age-dependent changes in testing rates compared to positivity rates. Further investigation of testing in relation to confirmed cases in children (Fig. 6A) and adults (Fig. 6B) shows a similar evolution over time, although with more variability in the number of tests per 100,000 children compared to adults (Figure S4). Overall test positivity rates in children are higher compared to those in adults (Figure S5).

### Reporting effectiveness

**Fig. 7:**
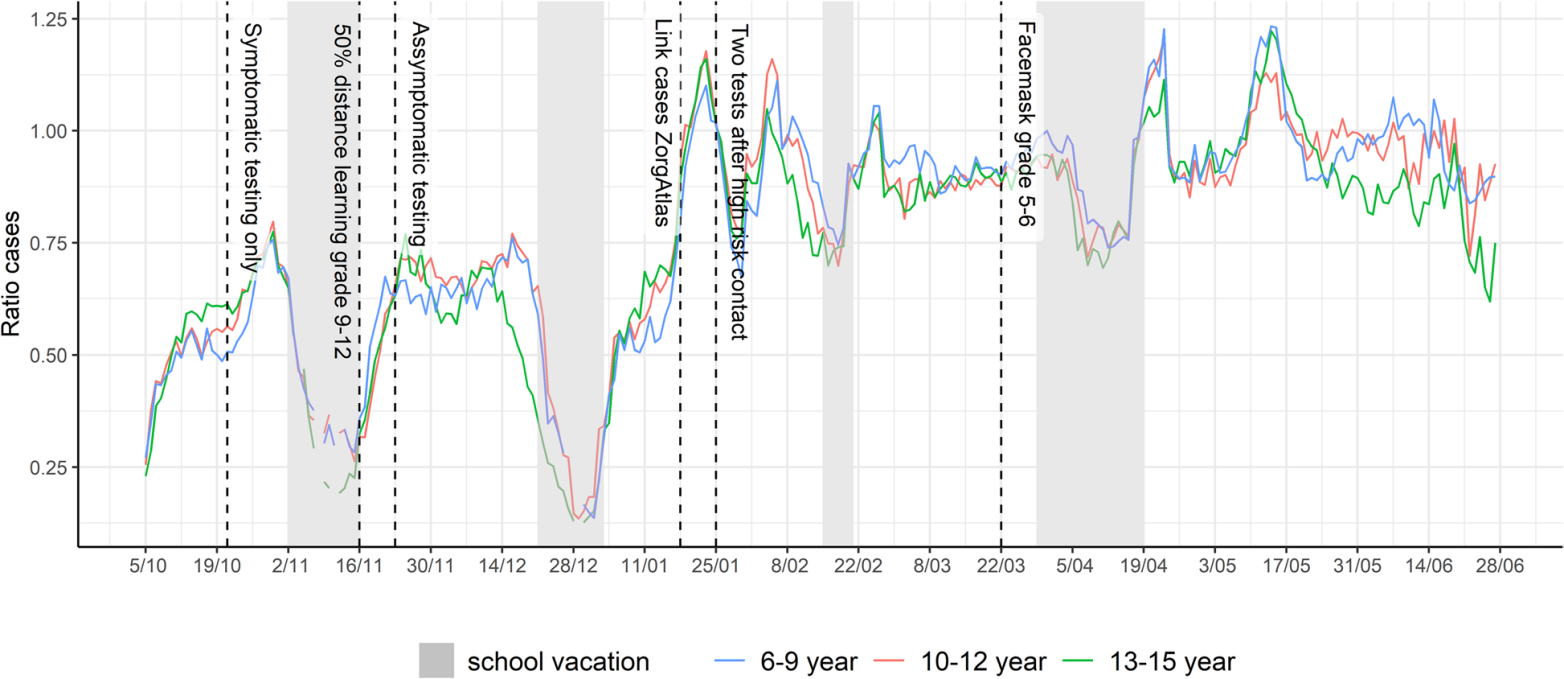
Reporting effectiveness: Moving window ratio cases captured in the surveillance network/Sciensano test-positive cases in children 6–15 years old. Pre-school: age 2.5-5yo, grade 1-2: 6&7 yo, grade 3-4: 8&9 yo; grade 5-6: 10&11 yo; grade 7-8: 12&13 yo; grade 9-10: 14&15yo; grade 11-12: 16&17yo; yo: years old, intended age of pupils on January 1th while in the corresponding grade

Holiday periods and changing test strategies are visually (Figs. 1 and 2) associated with shifts in case reporting. Cases captured in the surveillance system are equally dependent on holiday periods when compared with the nationally reported number of positive tests (Fig. 7), with a 60% reporting rate during non-holiday periods. Only ¼ of cases were reported during the first holiday periods of the academic year. Essentially complete reporting in the surveillance system was achieved after automated linkage and streamlined reporting of cases between the public health laboratory surveillance and the school health network, by exporting the laboratory confirmed cases directly into LARS after January 18^th^, 2021. Some limited reporting lag continues to occur, though this is uncommon due to nearly real-time reporting of most laboratories to the central COVID-19 testing database.

## Discussion

We describe the evolution of the COVID-19 epidemic in a large Belgian region where schools have been reopened and remained open almost the entire school year 2020–2021, using data from a newly developed school network-based surveillance program. We show that it is feasible to set up a surveillance network within the present structures of the school and school health network when a digital platform is available. We provide a descriptive analysis of the Flemish region school COVID-19 surveillance program, with presentation of age-specific data.

Age is one of the most important predictors of morbidity and mortality among those with COVID-19 [22–24]. The presence and importance of age-dependent susceptibility and transmission remain unclear, even more so with the change in dynamics with the spread of SARS-CoV-2 variants of concern, with increased transmission rates in all ages after the appearance of the alpha variant and with further increasing transmissibility among those infected with the omicron variant [25], including in children [26]. In addition, there is developing knowledge on the effect of sustained high and boosted vaccination coverage in the adult population [27], while the pediatric population has either substantially lower vaccine coverage (5–11-year-old) or still lacks an authorized vaccine (under 5 years old). In Flanders, SARS-CoV-2 vaccination was introduced during the last trimester of the reporting period of this study in the Flemish adult population. Two-dose coverage reached 44% in the > 18-year-old population at the end of the study period, however this figure includes 79% in the prioritised 65 + population at a moment of low virus circulation, not suspected to have yet an impact on the transmission dynamics in school-aged children at any point of the study. Vaccination in 12–18-year-old children was initiated only at the end of the academic year, and authorization for the under 12 population dates from December 15^th^, 2021, in Belgium [28].

The positive association between age and number of cases at the start of the school year in this study appears, at least temporally, to have disappeared, through a combination of age-specific NPIs, and NPI and age-dependent testing rules. A decrease in cases in grade 11–12 after the shift to 50% education in November 2020, and a later decrease in cases in grade 5–6 after the introduction of face masks in March 2021 were observed. The figures show the sequential overtaking of younger age groups with the largest number of cases per 100,000 population after the initiation of the grade specific interventions in the older groups, negating the age-dependent trends in case counts that were present at the start of the academic year and thereby closing the age-differences in susceptibility and infection. Mask wearing was previously introduced in all staff and pupils from grade 7 and up early in the 2020–2021 academic year. Studies investigating the effect of mask mandates [29] estimate a lower incidence rate ratio in districts with mask mandates for all pupils 2-year-old and up and a decrease in incidence after mask wearing introduction. Formal comparison studies between regions with differing mask mandates can build on this evidence base. During the 2021–2022 academic year, in the fall of 2022, a large fourth wave hit Flanders and universal masking in schools was introduced in grade 1 and up and this NPC introduction can be the basis of further work using the data of the continued surveillance network. We find no statistically significant difference between cumulative cases from grade 5–6 through grade 9–10 after March 2020 for the 2020–2021 academic year, our study period.

We show that testing has been very heterogeneous over time during the epidemic in the pediatric population, which is different from testing dynamics within the adult population. Testing regimens influence the tests per population performed more in the pediatric than the adult population [30]. Changing testing strategies, linked to contact risk stratification, in addition to decreased incentives to get testing for children while not at school, i.e., during school vacations or distance learning, provide missed opportunities for a more complete capturing and continuous follow up of the epidemic evolution in school aged children. Rules for assignment of high-risk versus low-risk contacts have implications for further testing, evaluation and quarantine. They directly affect case investigation and detection in the affected pupils. Adherence to these procedures therefore further affects and feeds the case finding and surveillance data, though without the possibility of having the ultimate complete data set and numerator of the truly infected. Our data thus far do not allow an unbiased estimate of the impact of testing regimens on the age-specific proportion of detected cases versus those undiagnosed [31], nor can we quantify, in this sample, the impact of testing as a sole NPI. However, without the timely HRC identification, quarantine, school-initiated testing, diagnosis and isolation of positive cases, which is made possible through the integration of the school surveillance system in the public health framework, it is likely that additional cases would have remained undiagnosed, thus allowing continued spread of the virus. The high-quality tracing activities of the CBL staff, with their ability to prescribe additional testing, contributed substantially to the efficiency of the general contact tracing, in addition to feeding the surveillance system. We cannot, however, completely describe the administrative hurdles experienced in the set-up and maintenance of the surveillance system by the responsible agencies. The burden on the CLB clinical and general staff corps has been high. It needs to be recognised that time and energy investment in surveillance systems focused on one infectious disease divert time and effort from other core activities oriented towards supporting the broader health in the school-aged population.

A stark finding is that pupils from schools with the lowest SES carried a quantified and important higher burden of COVID-19 over the 2020–2021 school year in our study. Flanders has no large network of private schools and the NPIs equally apply in all schools, however, the differences in cumulative incidence show how social characteristics of pupils as part of social arrangements in society also in the Flemish region differentially shape infectious disease dynamics and result in unequal disease burden. Data on disparities in the burden of COVID-19 in the general population have been published [32–34], with ethnic/racial minorities or historically racialized groups carrying a higher infection, disease and mortality burden and interactions of race/ethnicity with low SES through pathways of occupation and living conditions, including inability to isolate, resulting in overrepresentation in the COVID-19 case counts. Schools and pupils mirror our society and investigating the risk factors for infection and disease should include the assessment of the role of social determinants of health [33, 35], independent and specifically for the pediatric population [36, 37]. The pandemic clearly illustrates how this infectious disease pandemic is equally a social pandemic and implies that we need to provide additional support to the most vulnerable children and families.

The found differences in cumulative incidence by province reflect the heterogeneous provincial distribution of community cases. Even in the densely populated and well-connected region of Flanders, SARS-CoV-2 infection rates show a local distribution [38], dependent on heterogeneous mixing patterns and subject to different policies [39, 40]. Research shows that transmission in schools depends on the level of community transmission [41, 42]. School cases show a high correlation with the evolution of community cases in the general population in our study (i.e., Fig. 4).

There are multiple lessons learned from the implementation of the surveillance system and the analysis of its collected data. To improve data quality and to minimize the missingness of important variables, the introduction and inclusion of surveillance within an existing digital platform has proven to be crucial given collection of data by use of excel documents is insufficient, mainly to capture meta-data correctly. Variable definition can lead to unintended loss of information, for example, while the clinicians from the CLB’s gathered data on the presence of symptoms in tested individuals, this was not reflected in the data collection where only a single reason for testing (symptoms or HRC) could be entered. Hence, the reason of testing could not directly be used to assess the impact symptoms play in this pediatric population on testing and case detection. Nor was it possible to, with minimal risk for misclassification, calculate the secondary attack rate (SAR) in schools, an estimate of interest [43]. Further adaptations have already been implemented, including the use of linked laboratory surveillance system collected data, will allow analyses of outbreak size, SAR and reporting of proportion of symptomatic cases for following surveillance periods. We address underascertainment of cases through the automatization of the system, which resulted in a detectable increase in reporting efficiency. We cannot, however, sufficiently assess reporting delays in our analysis and formally quantify timeliness, nor its change over time, and assess the facilitating or delaying determinants. Weekend days and vacation periods suspectedly are suspected to lead to reporting delays, which we observed (Fig. 7), however such delays have decreased in duration following linkage of the surveillance system in January 2021.

The school environment of course does not only contain school children. The close interaction with the adult teaching and support staff has been the main concern for keeping the schools open, once the relatively low direct impact on morbidity [44, 45] and extremely low mortality [46] in the pediatric population became evident. The joint collection of cases in staff and pupils undoubtedly would add beneficial data, however this was shown complex and mainly burdened by administrative and other hurdles in this sample.

Availability of surveillance data serves multiple purposes [47]. The surveillance data described in this study are presented biweekly to the Ministry of Education and also included in the Sciensano weekly updates [20, 21]. Surveillance programs can detect and follow the evolution of registered cases in the school environment and provide baseline and follow-up data that can be compared to community surveillance data.

Suggestions to improve the data collection tool are the following: Addition of data of school absenteeism [48, 49], inclusion of total number of children tested (including data on test negatives), separate collection of the variable capturing the presence or absence of symptoms and the opportunity to report additional instituted interventions during a class or school outbreak for later evaluation of its impact on the size and time to containment of the outbreak. The surveillance network can be used for follow-up and to perform impact evaluations [50] of changes in school level interventions, like e.g. shifts in in-person education, changes in mask mandates, test modalities (antigen versus PCR) used for outbreak investigations, and others. With the availability of high-quality data, predictive models can be developed as alarm systems informing on the epidemic evolution and including calculation of the (changes in) SAR. Estimation of the attributable effect of interventions on the change of cases can additionally provide evidence relevant for policy decisions. In the future, one can also foresee that information on vaccination coverage and other details on circulating SARS-CoV-2 variants would be valuable for further study and should also be included in subsequent analyses.

## Conclusion

Establishing a surveillance system for a new infectious disease within a school health system is feasible and provides useful data on the evolution of the pandemic in the school environment. A school surveillance system for recurrent and new infectious diseases, which is automated and embedded in a structural manner within the public health system will improve epidemic response and can more swiftly provide epidemiological and scientific insights.

In the Flemish region associations between age and incidence case rates dissolved, hypothetically mitigated through age-specific school-level interventions, though potentially residually biased by age and intervention dependent changes in testing strategies. Differences in incidence by SES, however, remained.

## Supplementary Information

**Additional file 1: Supplement Figure 1.** Flow chart Surveillance network and Study sample. **Supplement Figure 2.** Surveillance system data flow and components. **Supplement Figure 3.** Relative difference in reported COVID-19 cases per week per 100,000 by grade relative to grade 7–8. **Supplement Figure 4.** Evolution number of confirmed SARS-CoV-2 PCR and antigen tests (left Y-axis) and positive tests (right Y-axis) in children 6-18 years old and in adults 19-79 years old in Flanders. **Supplement Figure 5.** SARS-CoV-2 test positivity rate adults 19-80 compared to children 6-18 in the community.

## Data Availability

The datasets used and/or analysed during the current study are available at the aggregate level from the corresponding author on reasonable request.

## Abbreviations

CLB: Centrum voor Leerlingenbegeleiding; Student Guidance Centers
CI: Confidence interval
COVID-19: Coronavirus disease of 2019
HRC: High-risk contact
IP: Incidence proportion, cumulative population incidences, incidence rates
LARS: Leerling Activiteiten en Registratie Systeem
NPIs: Non-pharmaceutical infection prevention and control interventions
PCR: Polymerase chain reaction
SAR: Secondary attack rate
SARS-CoV-2: Severe acute respiratory syndrome coronavirus 2
SES: Socioeconomic status
VAZG: Vlaams Agentschap Zorg en Gezondheid
