# Supplementary material for: COVID-19 surveillance in the Flemish school system: development of systematic data collection within the public health school system and descriptive analysis of cases reported between October 2020 and June 2021
Source: BMC Public Health. 2022 Oct 15;22:1921. doi: 10.1186/s12889-022-14250-1 (PMC9568939; doi:10.1186/s12889-022-14250-1)
Supplement: Supplementary file 1 — Additional file 1: Supplement Figure 1. Flow chart Surveillance network and Study sample. Supplement Figure 2. Surveillance system data flow and components. Supplement Figure 3. Relative difference in reported COVID-19 cases per week per 100,000 by grade relative to grade 7–8. Supplement Figure 4. Evolution number of confirmed SARS-CoV-2 PCR and antigen tests (left Y-axis) and positive tests (right Y-axis) in children 6-18 years old and in adults 19-79 years old in Flanders. Supplement Figure 5. SARS-CoV-2 test positivity rate adults 19-80 compared to children 6-18 in the community. [file 12889_2022_14250_MOESM1_ESM.docx]

**Supplementary Materials :**

### 1. Supplement Figure 1: Flow chart Surveillance network and Study sample:

Publicly funded Flemish school-networks:


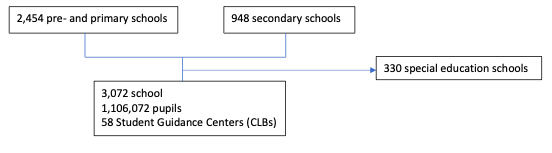


### 2. Supplement Figure 2: Surveillance system data flow and components

### 3. Supplement Figure 3:

Ratio reported cases per week per 100,000 relative to grade 7-8


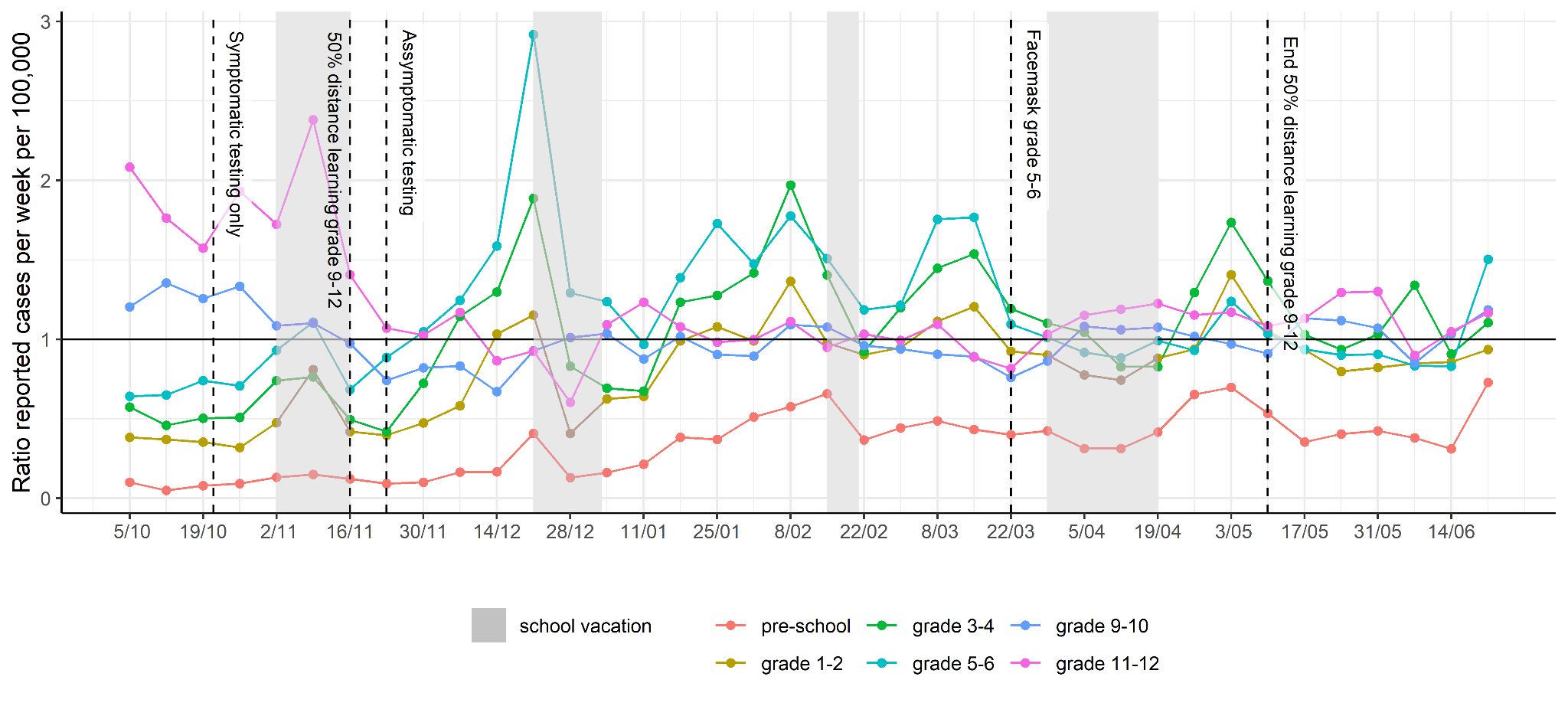


### Supplement Figure 4

Evolution number of confirmed SARS-CoV-2 PCR and antigen tests (left Y-axis) and positive tests (right Y-axis) in children 6-18 years old and in adults 19-79 years old in Flanders


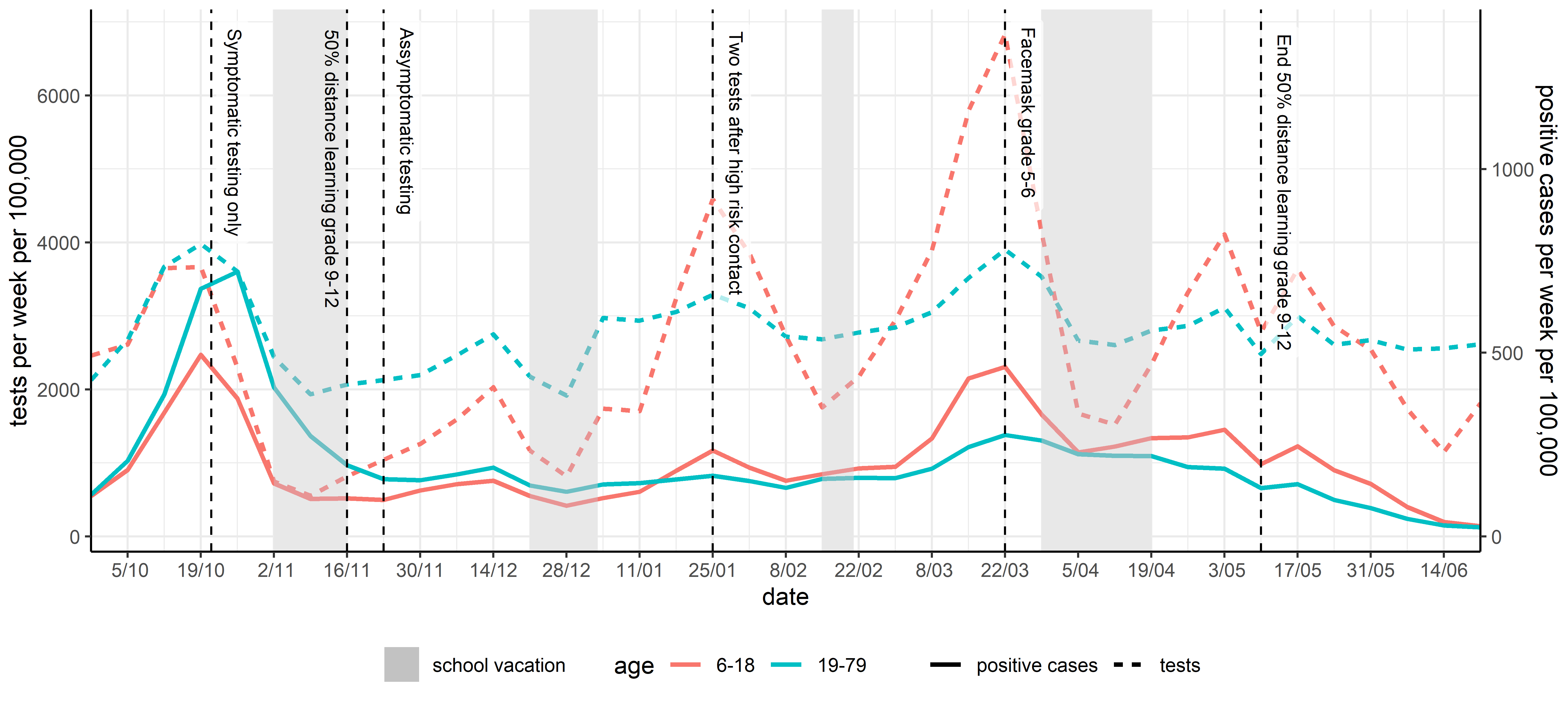


### Supplement Figure 5

SARS-CoV-2 test positivity rate adults 19-80 compared to children 6-18 in the community


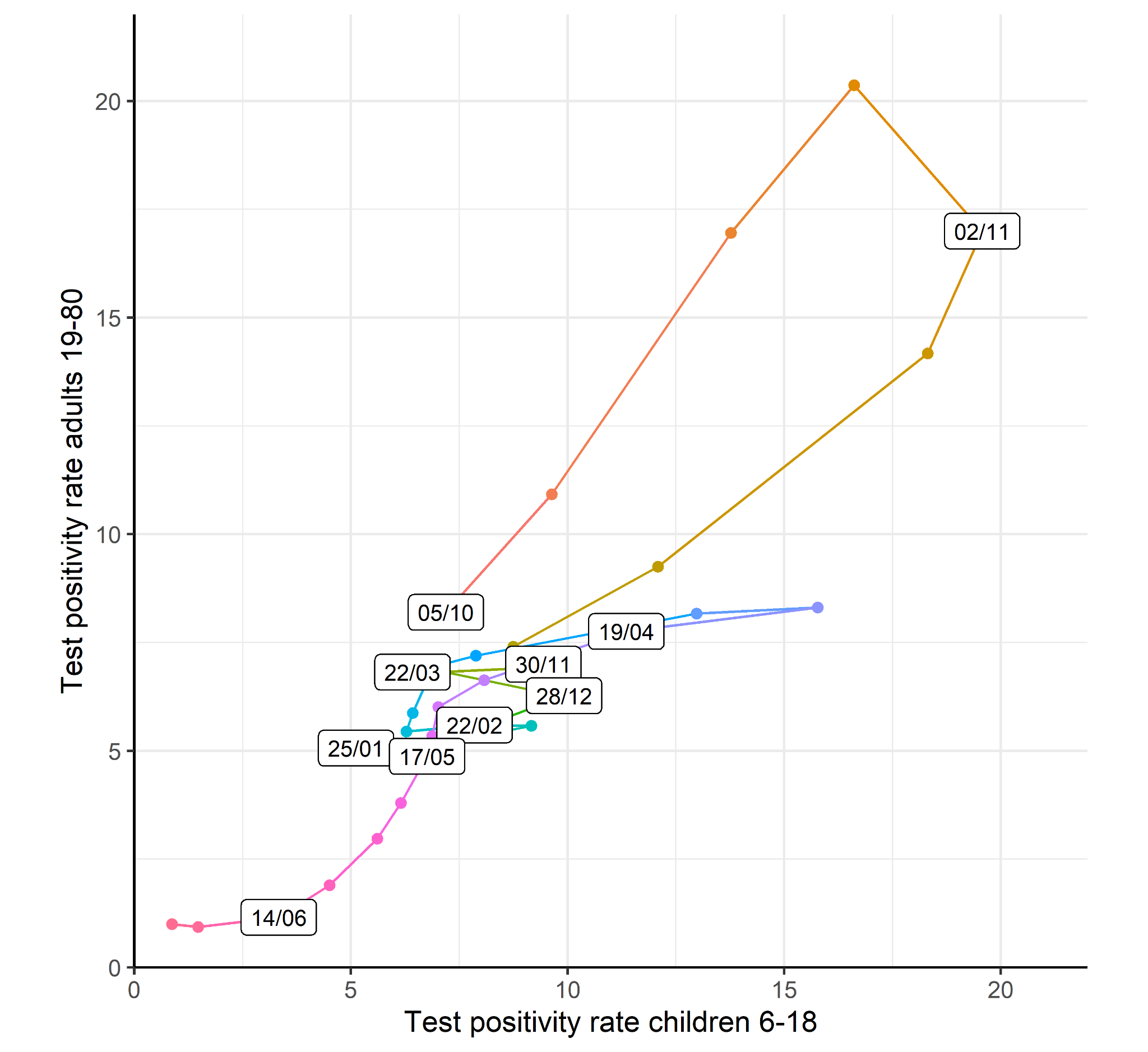


1. Supplement Non-Pharmaceutical Infection and Prevention Interventions with relation to the school network - additional details.

Source: Sciencano.

2020-10-23: Special educational activities outside school indoor limited to 40 persons. https://www.premier.be/nl/covid-19-alarmniveau-4-overlegcomit%C3%A9-verstrengt-coronaregels-voor-sport-cultuur-en-hoger-onderwijs

2020-10-28: School excursions forbidden in Brussels Capital. <https://www.bruxelles.be/sites/default/files/bxl/Arrete_de_police_bruxellois_-_2020.10.26_-_mesures_COVID_complementaires_.pdf>

2020-11-09: School activities outside school are restricted to the minimum for children (exception for sport and swimming), forbidden for adults

2021-02-08: One week online learning for all secondary school children in Flanders. <https://onderwijs.vlaanderen.be/nl/nieuws/update-corona-duidelijkheid-over-voorstel-krokusvakantie-en-over-mondmaskers-in-basisonderwijs>

2021-03-15: School activities outside school are allowed a maximum of 1 day per week. https://www.info-coronavirus.be/nl/news/occ-0503/

2021-03-15: Vulnerable Children in special needs education and vocational education are allowed 100% in person education.

2021-03-22: Extra school measures agreed by the regions (closure cafeteria, separating classes at playing time, pupils must avoid public transport and exclusion of situations of adult interactions in the school environment. https://www.info-coronavirus.be/nl/news/occ1903/

Procedures Sciensano: <https://covid-19.sciensano.be/nl/procedures/contactopvolging-kinderopvang-en-onderwijs>

Guidelines education Flemish region:

https://onderwijs.vlaanderen.be/nl/coronavirus/coronavirus-2021-2022
